# Noninvasive evaluation of neutrophil extracellular traps signature predicts clinical outcomes and immunotherapy response in hepatocellular carcinoma

**DOI:** 10.3389/fimmu.2023.1134521

**Published:** 2023-07-13

**Authors:** Hongjie Xin, Qianwei Lai, Yuchen Zhou, Jian He, Yangda Song, Minjun Liao, Jiarun Sun, Meng Li, Mingxia Zhang, Weifang Liang, Yang Bai, Yongyuan Zhang, Yuanping Zhou

**Affiliations:** ^1^ Department of Gastroenterology, Nanfang Hospital, Southern Medical University, Guangzhou, China; ^2^ Department of General Surgery, Cancer Center, Integrated Hospital of Traditional Chinese Medicine, Southern Medical University, Guangzhou, China; ^3^ Department of Infectious Diseases, Nanfang Hospital, Southern Medical University, Guangzhou, China; ^4^ HBVtech, Germantown, MD, United States

**Keywords:** hepatocellular carcinoma, neutrophil extracellular traps, computed tomography, radiomics, prognosis, programmed death receptor-1

## Abstract

**Background:**

Neutrophil extracellular traps (NETs) have been shown to play a pivotal role in promoting metastasis and immune escape in hepatocellular carcinoma (HCC). Therefore, noninvasive tests to detect the formation of NETs in tumors can have significant implications for the treatment and prognoses of patients. Here, we sought to develop and validate a computed tomography (CT)-based radiomics model to predict the gene expression profiles that regulate the formation of NETs in HCC.

**Methods:**

This study included 1133 HCC patients from five retrospective cohorts. Based on the mRNA expression levels of 69 biomarkers correlated with NET formation, a 6-gene score (NETs score, NETS) was constructed in cohort 1 from TCIA database (n=52) and validated in cohort 2 (n=232) from ICGC database and cohort 3 (n=365) from TCGA database. And then based on the radiomics features of CT images, a radiomics signature (RNETS) was developed in cohort 1 to predict NETS status (high- or low-NETS). We further employed two cohorts from Nanfang Hospital (Guangzhou, China) to evaluate the predictive power of RNETS in predicting prognosis in cohort 4 (n=347) and the responses to PD-1 inhibitor of HCC patients in cohort 5 (n=137).

**Results:**

For NETS, in cohort 1, the area under the curve (AUC) values predicting 1, 2, and 3-year overall survival (OS) were 0.836, 0.879, and 0.902, respectively. The low-NETS was associated with better survival and higher levels of immune cell infiltration. The RNETS yielded an AUC value of 0.853 in distinguishing between high-NETS or low-NETS and patients with low-RNETS were associated with significantly longer survival time in cohort 1 (*P*<0.001). Notably, the RNETS was competent in predicting disease-free survival (DFS) and OS in cohort 4 (*P*<0.001). In cohort 5, the RNETS was found to be an independent risk factor for progression-free survival (PFS) (*P*<0.001). In addition, the objective response rate of HCC patients treated with PD-1 inhibitor was significantly higher in the low-RNETS group (27.8%) than in the high-RNETS group (10.8%).

**Conclusions:**

This study revealed that RNETS as a radiomics biomarker could effectively predict prognosis and immunotherapy response in HCC patients.

## Introduction

Hepatocellular carcinoma (HCC), the most commonly occurring primary liver cancer, remains a global health challenge and is the third leading cause of cancer-related death worldwide (1). The tumor immune microenvironment (TIME), which is composed of various tumor-infiltrating immune cells, is thought to greatly affect tumor progression and response to immunotherapy (2). Currently, blocking the signaling of the programmed cell death receptor-1 (PD-1) and programmed cell death receptor ligand-1 (PD-L1) pathways with monoclonal antibodies is a new immunotherapeutic approach for treating advanced HCC cases. However, the response rates to this treatment have been low and the overall prognosis for HCC patients does not seem to improve with this treatment, probably because of the TIME heterogeneity (3–5). Therefore, tools that can accurately determine the TIME status could be invaluable in guiding treatment for HCC patients.

Neutrophils that infiltrate tumors, also called tumor-associated neutrophils (TANs), often play pro-tumorigenic roles and support tumor progression in response to microenvironmental cues released by tumor and stromal cells (6, 7). Neutrophil extracellular traps (NETs), which are extracellular web-like structures of DNA chromatin complexes extruded from dying neutrophils, were once thought to mainly function as snares that caught and killed harmful microorganisms (8). There is increasing evidence to prove that in cancers, NETs play important role in promoting metastasis (9–11). Recent analyses suggested that studies on NETs are becoming increasingly momentous in research on liver cancer. For example, one study indicates that the elimination of NETs may slow the progression of steatohepatitis-related liver cancer (12). In addition, the formation of NETs is known to trigger pro-tumorigenic inflammatory responses and fuel HCC metastasis (13). Furthermore, NETs have been reported to protect cancer cells from being attacked by the immune system and reduce the effectiveness of immunotherapy (14–16). Thus, scoring model that can evaluate the formation potential of NETs in tumors can be useful as biomarkers for predicting survival and in estimating if immunotherapy could be of benefit to HCC patients.

By using the expression profiles of genes associated with NETs formation in biopsy specimens, we are able to directly measure the abundance of NETs in tumor tissues. Recent studies have shown that the integration between machine learning algorithms and NETs-related gene signatures own the potential to be reliable biomarkers in predicting prognosis and the response to immunotherapy in several types of malignant tumors (17, 18). However, this method has some major limitations, including the requirement for an invasive procedure and a forbiddingly high cost for patients. The emergence of radiomics, which uses quantitative medical imaging features, maybe a viable alternative to the method mentioned previously (19, 20). Computed tomography (CT) is now in standard use for diagnosing, staging and monitoring therapeutic efficacy in liver tumors. CT-based radiomics have shown that it is capable of achieving successful assessment and predictive ability in prognostic aspect for HCC patients (21–23). Given the increasing use of immunotherapy in advanced HCC cases, the role of CT radiomics in characterizing TIME necessitates more exploration and amelioration. Some research has shown that certain radiological features of tumors are closely related to specific gene expression profiles; this provides a unique opportunity for developing non-invasive complementary approaches to detect NETs in the TIME (24, 25).

This study has three objectives. One, to identify a NETs-related gene signature (NETs score or NETS) that correlated with patients’ prognosis and could differentiate ‘cold’ or ‘hot’ TIME. Two, to establish a link between the NETs score and tumor radiomics features obtained from contrast-enhanced CT. And three, to build a novel radiomics model (radiomics NETs score or RNETS) to predict survival and to identify HCC patients for whom immunotherapy would be beneficial.

## Materials and methods

### Study design and data collection

**Figure 1:**
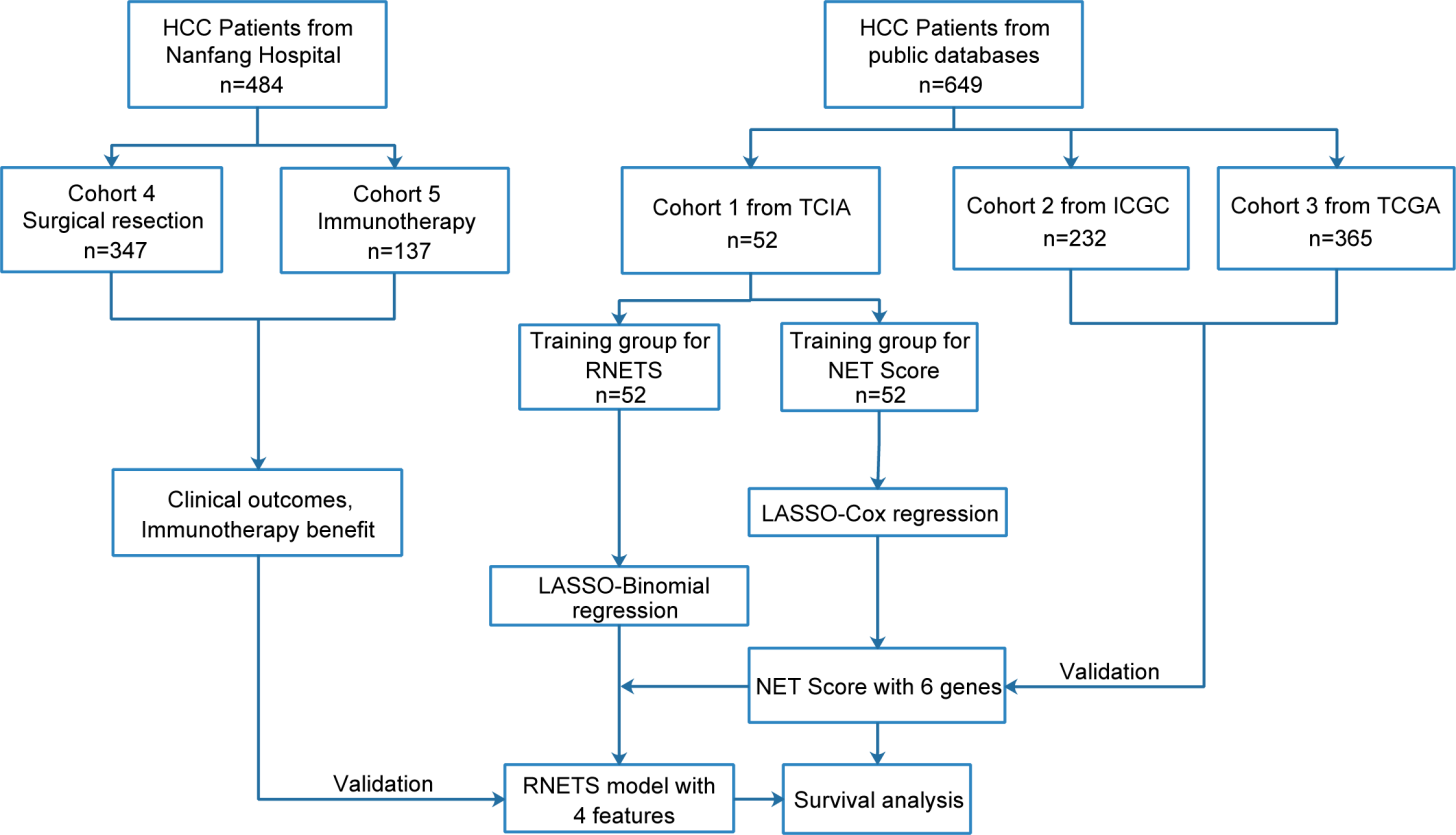
Flowchart of overall study.

This study retrospectively included five cohorts and the flowchart in **Figure 1** describes the entire study design. The HCC patients with RNA sequence data, baseline CT images and the corresponding clinical data from The Cancer Imaging Archive (TCIA, n=52) were collected as training cohort (Cohort 1) to firstly develop a prognostic NETs-related gene signature (NETs score, NETS), and then construct a CT-based radiomics model (radiomics NETs score, RNETS) that correlated with expression patterns of NETS by using machine learning algorithms. Two external validation cohorts with RNA sequence data and complete follow-up data were downloaded from International Cancer Genome Consortium (ICGC, n=232, Cohort 2) and The Cancer Genome Atlas (TCGA, n=365, Cohort 3) to validate the prognostic value of NETS. Besides, HCC patients with baseline CT information from Nanfang Hospital (Guangzhou, China) who underwent curative resection between January 2013 and October 2018 (Cohort 4, n=347) were used to evaluate the predictive value of RNETS in predicting the risk of postoperative recurrence, while those receiving immunotherapy with unresectable HCC between January 2019 and October 2021 (cohort 5, n=137) from Nanfang Hospital were used to evaluate the predictive power of RNETS in predicting the response to immunotherapy.

For patients without previous treatment, CT imaging data at baseline were collected for RNETS establishment and evaluation. Besides, for patients who were refractory to the standard first-line therapy (loco-regional treatments or targeted therapies) and received immunotherapy as second-line treatment, imaging data for analysis were collected based on the inclusion criteria as follows: 1. available CT imaging data within 30 days prior to PD-1 inhibitors therapy; 2. with ≥ 1 measurable target lesion. Follow-up data were collected from the initiation of treatment until 31 October 2022. Disease-free survival (DFS) was defined as the time from surgery to tumor recurrence at any site or death from any cause. Progression-free survival (PFS) was set to the time period from the execution of immunotherapy to disease progression or death from any cause. The responses to PD-1 inhibitors were defined as partial response (PR), complete response (CR), stable disease (SD), or progressive disease (PD), and evaluated by CT or magnetic resonance imaging (MRI) at 3 months and 6 months according to the RECIST version 1.1 (26).

### Establishment and evaluation of NETs score

A total of 69 NETs-related genes were collected from previous studies (8, 27) and are shown in **Table S1**. Cox proportion hazards regression with the least absolute shrinkage and selection operator (LASSO) was applied to tune the optimal value of the penalty parameter (λ) and derive the genes with the highest predictive value from training cohort with non-zero coefficients identified. The calculation formula for NETs score is presented as follows:


$$f\left(x\right)=\sum_{i=1}^{n}\left(P_{i}\beta_{i}\right)$$

Where n, P, and β denoted the number of selected genes, the normalized gene expression level, and the coefficient index derived from LASSO regression, respectively. The expression level was obtained by log2-transformed FPKM+1 of each gene. The ‘survminer’ package was employed to pick out the cut-off point for the NETs score, according to which, patients were divided into two groups (high-NETS and low-NETS). Subsequently, Kaplan-Meier curves were plotted and the log-rank method was employed to compare the survival distributions of the two groups. Utilizing ‘pROC’ R package and ‘timeROC’ R package to output receiver operating characteristic (ROC) curves and evaluate the predictive value according to the area under the curves (AUC).

### Immune infiltration assessment

The CIBERSORT deconvolution algorithm was used to quantify the infiltration levels of 22 classes of immune cells in the high- and low-NETS groups (28). In this step, we analyzed the data of normalized gene expression values combined with the LM22 signature and performed 1,000 permutations in R studio (v4.1.0). ESTIMATE was used to calculate different scores to describe the immune microenvironment, such as the infiltration level of immune cells (ImmuneScore), and stromal content (StromalScore) (29). Box plots were generated based on the scores obtained by the two NETS groups.

### Functional pathway enrichment analysis

Differentially expressed genes (DEGs) between the high- and low-NETS groups were obtained by using the empirical Bayesian approach via the ‘limma’ package. The significance criteria for selecting the DEGs were set as the false discovery rate (FDR)< 0.05 and the absolute value of the log2 (fold change) was set to ≥ 1.5. We then performed Gene Set Enrichment Analysis (GSEA) using the ‘GSEAbase’ R package to recognize the hallmark pathways in low- and high-NETS groups respectively.

### CT image segmentation and radiomics feature extraction

The CT images from the portal venous phase were fetched for extracting image features. We made use of the ITK-SNAP software (v4.0) (www.itksnap.org) to perform the delineation for the regions of interest (ROIs), in which two radiologists (Reader 1 and Reader 2, both with > 5 years of experience) manually segment the entire tumor on each axial slice. The CT images were resampled to a voxel size of 1×1×1 mm^3^ to standardize the voxel spacing. Voxel intensity values were discretized by a fixed bin width of 25 hounsfield unit (HU) to reduce image noise and normalize intensities; wavelet filtering was employed to all the CT series.

Subsequently, 1316 radiomics features were extracted from each ROI using the Pyradiomics Python package (version 3.0). Seven types of features were included: 1) Shape; 2) First Order Statistics; 3) Gray Level Co-occurrence Matrix; 4) Gray Level Run Length Matrix; 5) Gray Level Size Zone Matrix; 6) Neighboring Gray Tone Difference Matrix, and; 7) Gray Level Dependence Matrix.

### Feature selection and RNETS score calculation

**Figure 2:**
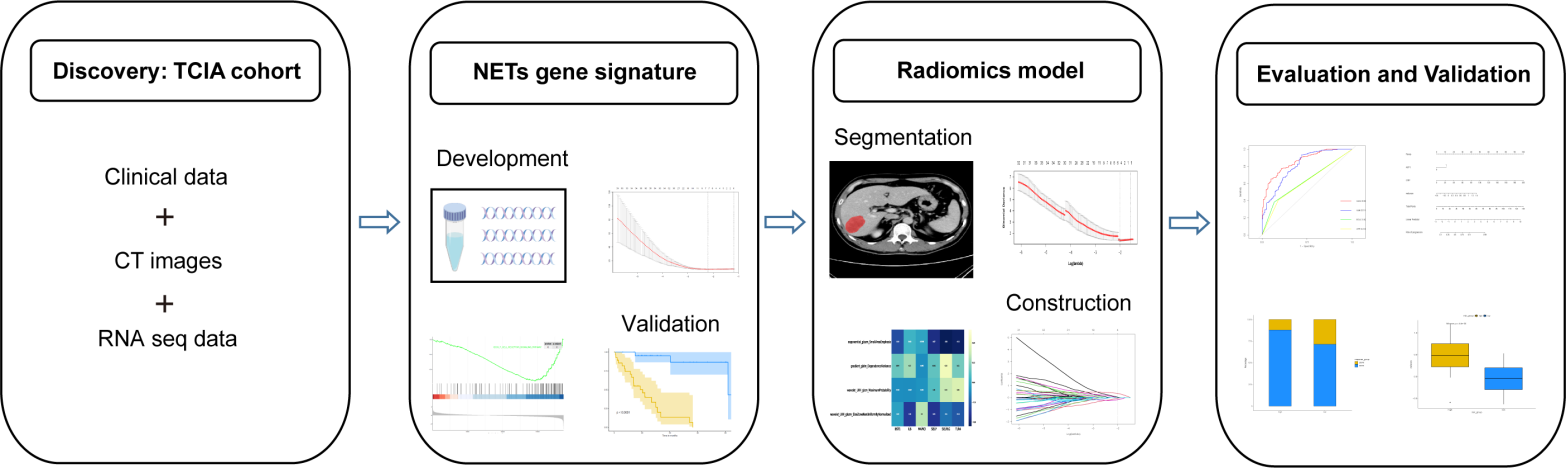
Detailed workflow of radiomics model (RNETS).

Before further analysis, to eliminate the differences in the value scales, we carried out a standardization process for all the extracted image features with z-scores. For feature selection, Spearman’s rank correlation analysis was performed and features with correlation coefficient values higher than 0.9 were considered to be redundant and would be filtered out. The LASSO regression was then employed to determine the features with the highest predictive value for the NETS with the penalty parameter tuning conducted by 5-fold cross-validation. The radiomics signature (RNETS) was constructed through a linear combination of the selected features weighted by their corresponding coefficients in the training cohort. The whole workflow of radiomics model is depicted in **Figure 2**.

### Statistical analysis

All statistical analyses and visualization plots were carried out in R studio (version 4.1.0, http://www.r-project.org) and SPSS 22. Correlations between continuous variables were estimated using the Spearman test. Comparisons between two or more group were carried out using either the Wilcox test or the Kruskal-Wallis test. The nomogram and calibration curves were established by using the ‘RMS’ package. All P values are two-sided and lower than 0.05 are regarded as statistical significance.

## Results

### Clinical characteristics

This study retrospectively included 1133 pathologically-confirmed HCC patients from five cohorts. Of these, 52 patients (Cohort 1) were from TCIA database [34 (65.3%) men and 18 (34.7%) women with mean age 61.02 ± 14.13 years], 232 patients (Cohort 2) were from ICGC database [171 (73.7%) men and 61 (26.3%) women with mean age 67.25 ± 10.11 years], 365 patients (Cohort 3) were from TCGA database [246 (67.4%) men and 119 (32.6%) women with mean age 59.65 ± 13.34 years]. In addition, among these patients from Nanfang Hospital (Guangzhou, China), 310 patients (89.3%) in Cohort 4 were male (mean age: 54.63 ± 11.21 years) and 123 patients (89.8%) in Cohort 5 were male (mean age: 50.82 ± 11.28 years). Of these 137 patients in Cohort 5, 39 (28.4%) received PD-1 inhibitors as first-line treatment, 98 (71.6%) received PD-1 inhibitors as second-line treatment who had undergone loco-regional treatments and targeted therapy (Sorafenib or Lenvatinib) prior to immunotherapy. More detailed clinical characteristics of these two Nanfang Hospital cohorts are listed in **Table 1**.

**Table 1:** Clinical characteristics in two Nanfang Hospital cohorts.

	Cohort 4 (n=347)	Cohort 5 (n=137)
Age (years), mean (SD)	54.63 (11.21)	50.82 (11.28)
Gender (%)		
Female	37 (10.7)	14 (10.2)
Male	310 (89.3)	123 (89.8)
Aetiology (%)		
HBV	298 (85.9)	129 (94.2)
HCV	21 (6.1)	1 (0.7)
Others	28 (8.1)	7 (5.1)
AFP (ng/ml), (%)		
≤ 400	236 (68.0)	56 (40.9)
> 400	111 (32.0)	81 (59.1)
ALBI grade (%)		
1	184 (53.0)	40 (29.2)
2	161 (46.4)	83 (60.6)
3	2 (0.6)	14 (10.2)
Child-Pugh grade (%)		
A	327 (94.2)	106 (77.4)
B	20 (5.8)	30 (21.9)
C	0 (0)	1 (0.7)
ECOG PS		
0	331 (95.4)	79 (57.7)
1 + 2	16 (4.6)	58 (42.3)
Cirrhosis		
Absent	116 (33.6)	26 (19.0)
Present	229 (66.4)	111 (81.0)
BCLC Stage (%)		
0	22 (6.3)	0 (0)
A	225 (64.8)	0 (0)
B	57 (16.4)	36 (26.3)
C	43 (12.4)	101 (73.7)
Embolus (%)		
Absent	313 (90.5)	52 (38.0)
Present	33 (9.5)	85 (62.0)
Tumor number (%)		
≤ 3	308 (89.0)	46 (33.6)
> 3	38 (11.0)	91 (66.4)
Tumor size (cm), median [IQR])	4.80 [3.10, 7.40]	9.20 [6.80, 12.70]
CRP (median [IQR])	/	18.19 [5.20, 28.60]
NLR (median [IQR])	/	3.08 [2.20, 3.71]
RNETS (mean (SD))	0.25 [0.00, 0.47]	0.21 (0.33)

AFP, α-fetoprotein; BCLC Stage, Barcelona Clinic Liver Cancer stage; ECOG PS, Eastern Cooperative Oncology Group performance status.

ALBI, albumin-bilirubin; CRP, C-reactive protein; NLR, Neutrophil-lymphocyte ratio.

### Development of scoring model based on NETs-related genes

**Figure 3:**
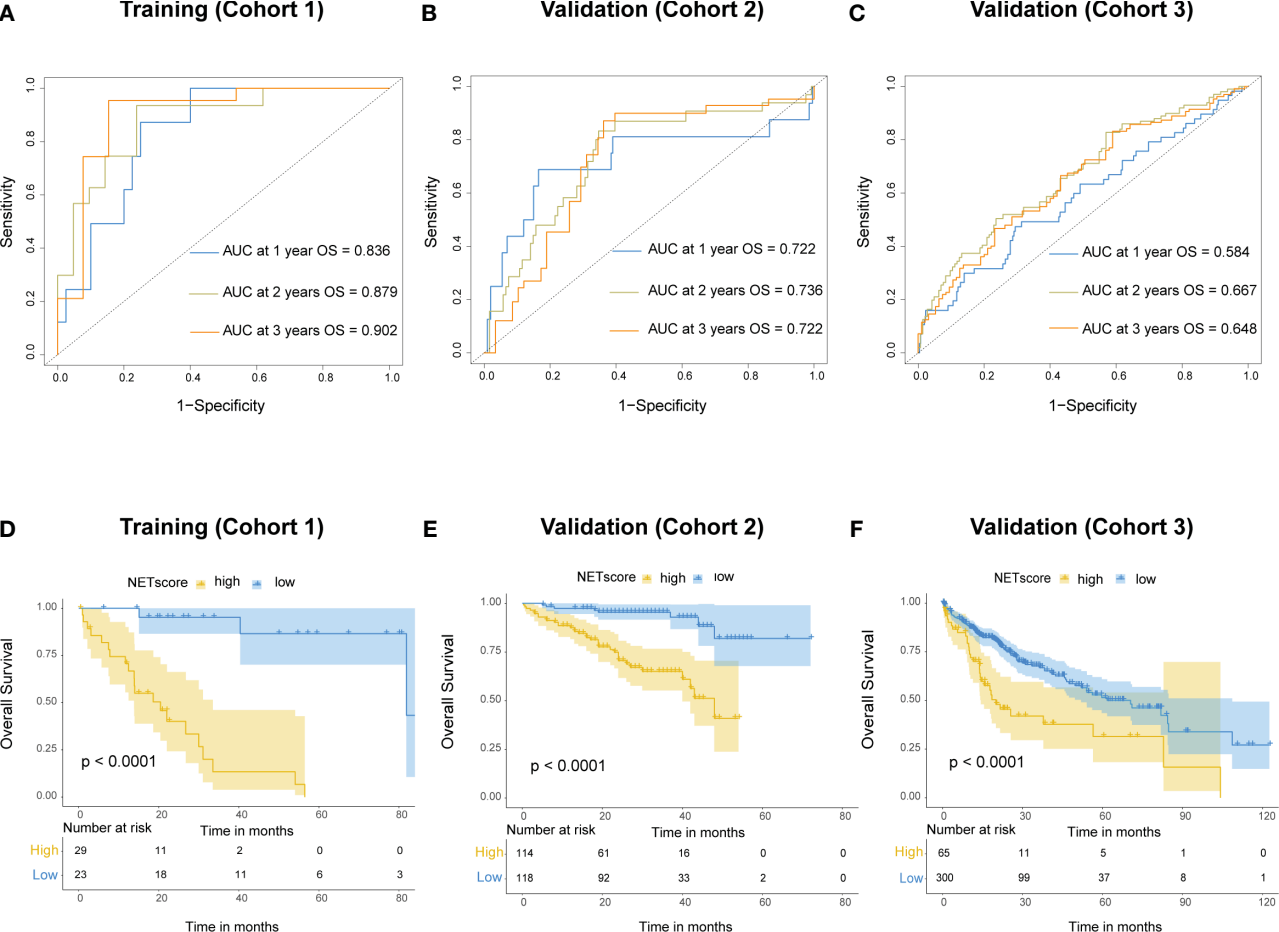
Prognostic value of NETs score. **(A)** Receiver operating characteristic (ROC) curves of NETs score for predicting overall survival (OS) at 1 year, 2 years, and 3 years, respectively in cohort 1, **(B)** cohort 2, **(C)** and cohort 3. **(D)** Kaplan-Meier curves of OS for patients with different NETs score in cohort 1, **(E)** cohort 2, **(F)** and cohort 3.

To better apply the NETs-initiation biomarkers to clinical management of HCC, these 69 NETs-related genes were further filtered by LASSO regression analysis to identify six hub genes with lambda.1se values of 0.3077334 (**Figure S1**). These six genes (*BST1*, *IL-6*, *MAPK3*, *SELP*, *SELPLG* and *TLR4*) were used to build a NETs-related risk signature called “NETs score”, which was calculated for each patients according to the following formula: NETs score = (-0.164×*BST1*) + (-0.055×*IL6*) + (1.419×*MAPK3*) + (-0.518×*SELP*) + (-0.714×*SELPLG*) + (-0.574×*TLR4*). The NETs score (NETS) was a good indicator for the prognosis of HCC patients in the training cohort, and the area under the curve (AUC) values were 0.836, 0.879, and 0.902 for 1, 2, and 3-year overall survival (OS), respectively (**Figure 3A**). The same calculation were performed in cohort 2 and cohort 3 as the validation dataset (**Figures 3B, C**). The best NETs score cut-off point of 0.8033756 was used to divide patients into high-NETS and low-NETS groups. The low-NETS group had a survival advantage over the high-NETS group in the training cohort (*P*<0.001) (**Figure 3D**). The similar results were also observed in validation datasets (cohort 2, *P*<0.001; cohort 3, *P*<0.001) (**Figures 3E, F**).

### Immune microenvironment heterogeneity underlying the NETS

**Figure 4:**
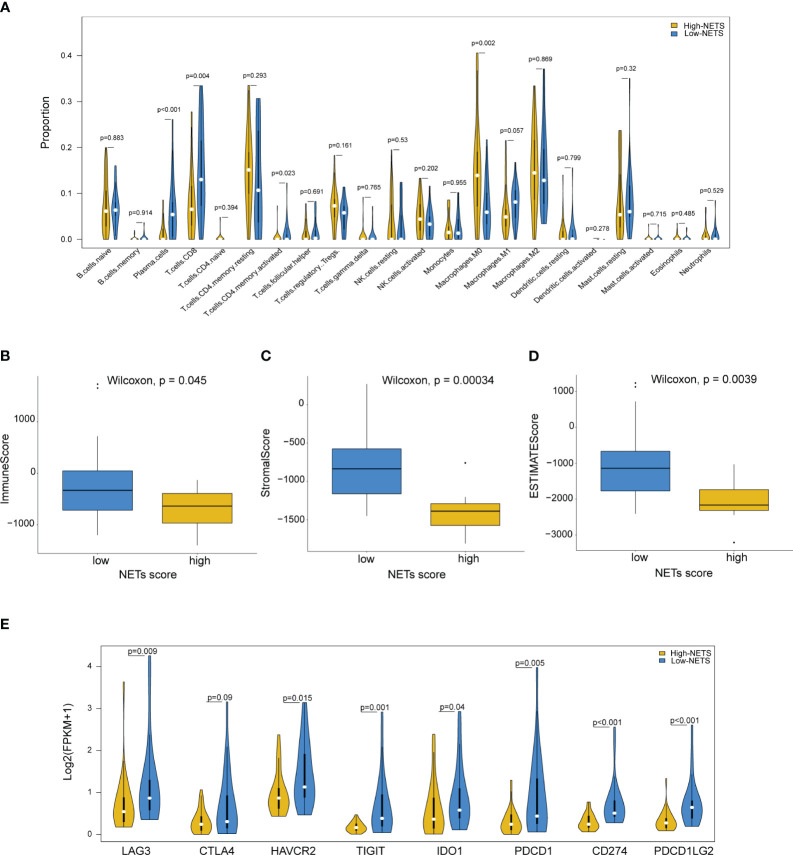
Diverse features of immune environment between NETS-H and NETS-L groups. **(A)** The infiltration levels of 22 immune cells between two groups analyzed by CIBERSORT algorithm. **(B-D)** The immune score, stromal score and estimate score analyzed in NETS-H and NETS-L groups, respectively. **(E)** The expression level of immune checkpoint genes between two groups. NETS, NETs score; NETS-H, high NETs score; NETS-L, low NETs score.

The results from the CIBESORT algorithm showed that immune-activated cells, including plasma cells, activated CD4 memory cells and CD8 T cells were significantly higher infiltration in the low-NETS group (**Figure 4A**). The ImmuneScore, StromalScore, and ESTIMATEScore values decreased as the NETs score increased (**Figures 4B-D**). Compared with the high-NETS group, the low-NETS group exhibited significantly higher expression levels of immune checkpoint genes (**Figure 4E**). Moreover, through GESA (**Table S2**), several immune response related pathways were also activated in the low-NETS group (**Figure S2**). In summary, these results suggest that an immunological ‘hot’ microenvironment exists in the low-NETS group, which tends to respond better to immunotherapy.

### Construction of a radiomics biomarker correlated with the NETS

**Figure 5:**
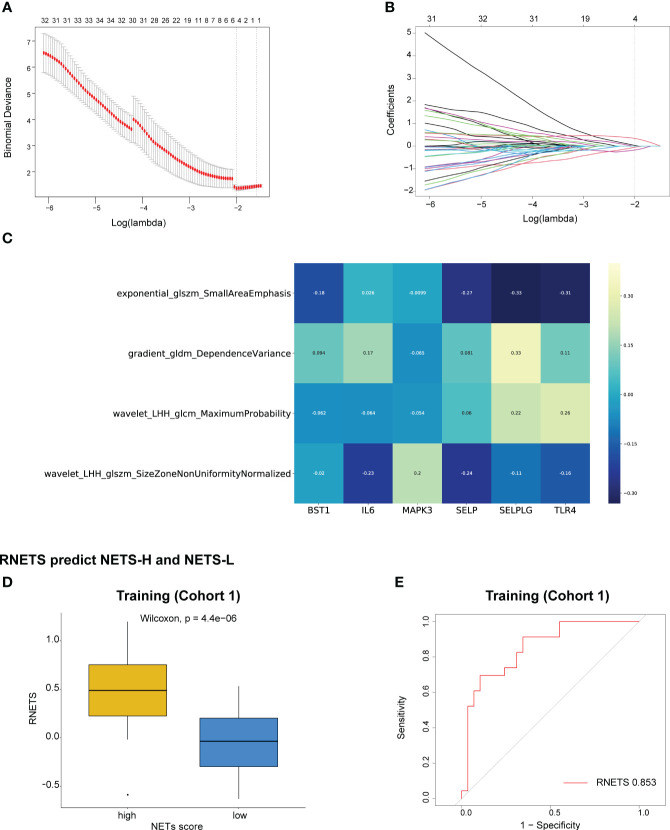
Construction of RNETS in training cohort. **(A)** Plot of tuning parameter log (λ) selection in LASSO model with 5-fold cross-validation. **(B)** Plot of coefficient profiles for all the radiomics features. **(C)** The correlation between the four radiomics features and six NETs-related genes. **(D)** The difference of RNETS between NETS-H and NETS-L groups. **(E)** The predictive value of RNETS for distinguishing NETS-H and NETS-L groups in cohort 1 (training). NETS, NETs score; NETS-H, high NETs score; NETS-L, low NETs score.

The Spearman correlation analysis was utilized to filter out redundant image features, following which, four radiomics features were eventually selected using the LASSO regression to construct a predictive radiomics biomarker for NETS status (high- or low-NETS) in the training cohort (**Figures 5A, B**). The association between the four radiomics features and six NETs-related genes were depicted in **Figure 5C**. The RNETS was significantly different between high- and low-NETS groups (*P*<0.001) (**Figure 5D**). The AUC value for distinguishing between high- and low-NETS was 0.853 in the training cohort (**Figure 5E**).

### Prognostic value of the radiomics biomarker

**Figure 6:**
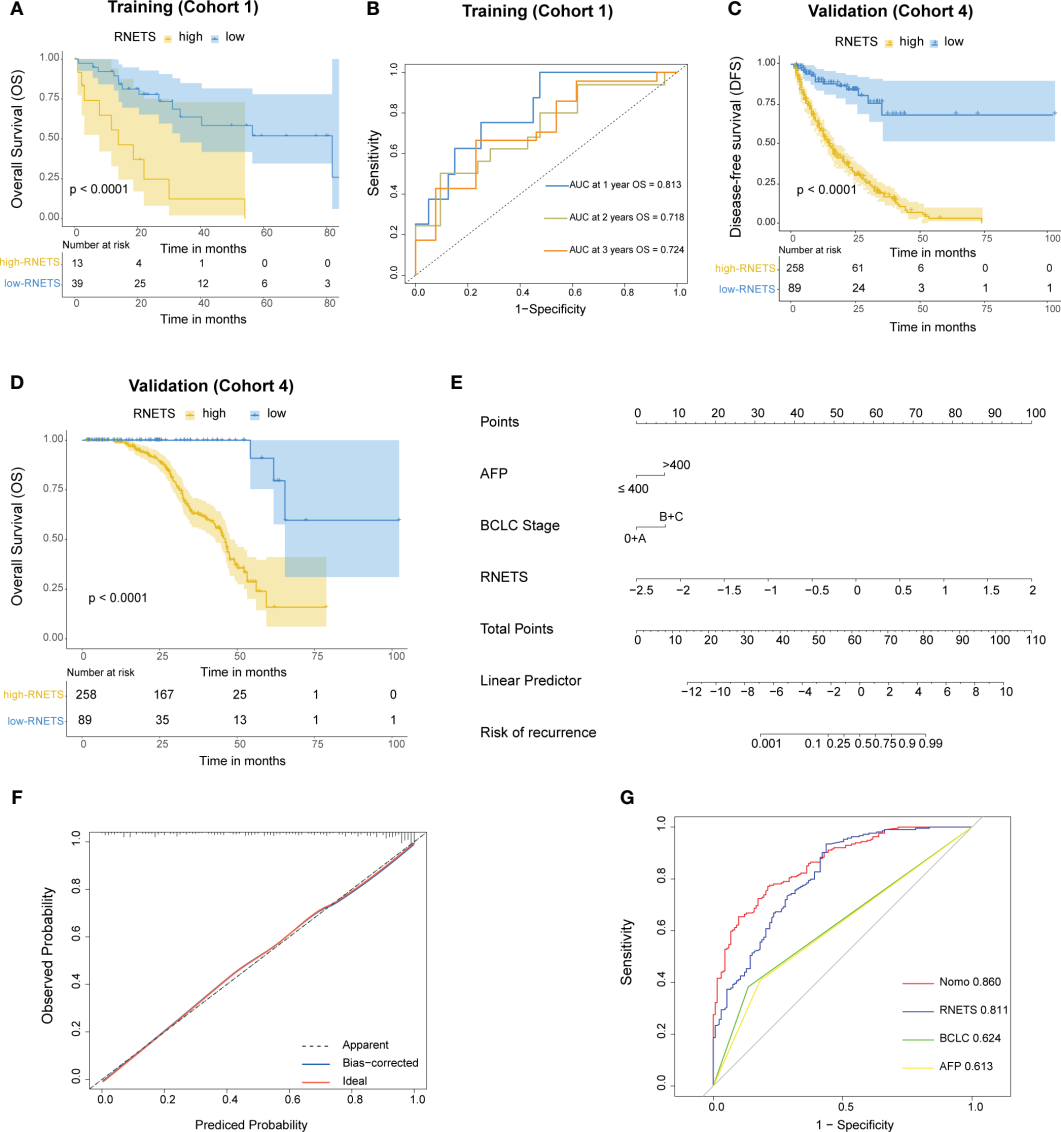
Prognostic value of RNETS. **(A)** Kaplan-Meier curves of overall survival (OS) for different RNETS groups in cohort 1 (training). **(B)** Receiver operating characteristic (ROC) curves of RNETS for predicting OS at 1 year, 2 years, and 3 years, respectively in cohort 1 (training). **(C, D)** Kaplan-Meier curves of disease-free survival (DFS) and OS for different RNETS groups in cohort 4. **(E)** Nomogram for predicting the risk of postoperative recurrence in cohort 4. **(F)** Calibration curves of the nomogram model. **(G)** ROC curves of recurrence status for the nomogram and three variables in cohort 4. AFP, a-fetoprotein; BCLC, Barcelona Clinic Liver Cancer.

The RNETS cut-off value of 0.8033756 was used to divide patients into high-RNETS and low-RNETS groups. Patients with low-RNETS were associated with significantly longer survival time in the training cohort (*P*<0.001) (**Figure 6A**). The AUC values of ROC curves were 0.813, 0.718, 0.724 for the 1-year, 2-year and 3-year OS, respectively (**Figure 6B**). The utility of the RNETS was further evaluated in the cohort. Kaplan-Meier curves showed that the low-RNETS group had significantly longer DFS and OS period as compared to those in the high-RNETS group in cohort 4 (*P*<0.001) (**Figures 6C, D**). Multivariate Cox regression analysis showed that the RNETS was an independent risk factor for postoperative recurrence. In addition, the BCLC stage and AFP levels were also significantly positively associated with the DFS in cohort 4 (**Table 2**). To broaden its clinical application, the variables were then integrated to develop a nomogram model (**Figure 6E**). The calibration curve showed there was a good consistency between the predicted value of recurrence and the actual observed value (**Figure 6F**). Importantly, in cohort 4, the AUC values of the nomogram for predicting DFS (0.860) were higher than those of RNETS, AFP and BCLC stage (**Figure 6G**), indicating that the nomogram has enhanced power in predicting recurrence as compared to RNETS, AFP levels or BCLC stage alone.

**Table 2:** Univariate and multivariate Cox regression analyses of risk factors for disease-free survival in Cohort 4.

	Univariate HR (95% CI)	*P* value	Multivariate HR (95% CI)	*P* value
AFP, ng/ml				
≤ 400	1.0			
> 400	1.76 (1.34-2.32)	<0.001	1.67 (1.26 - 2.22)	<0.001
Age	0.99 (0.98-1.01)	0.254		
ALBI grade				
1	1.0			
2	1.02 (0.77-1.33)	0.914		
3	1.4 (0.34-5.67)	0.641		
BCLC				
0+A	1.0			
B+C	2.54 (1.92-3.35)	<0.001	2.1 (1.58 - 2.80)	<0.001
Child-Pugh grade				
A	1.0			
B	0.95 (0.53-1.7)	0.852		
Cirrhosis				
Absent	1.0			
Present	1.02 (0.77-1.36)	0.868		
ECOG PS				
0	1.0			
1	1.56 (0.87-2.8)	0.138		
Embolus				
Absent	1.0			
Present	1.46 (0.96-2.24)	0.080		
Aetiology				
HBV	1.0			
HCV	0.52 (0.24-1.10)	0.089		
Others	0.76 (0.46-1.25)	0.280		
RNETS	2.81 (2.02-3.92)	<0.001	2.73 (1.94 - 3.83)	<0.001
Gender				
Female	1.0			
Male	0.88 (0.57-1.35)	0.558		
Tumor number				
≤ 3	1.0			
> 3	1.37 (0.9-2.08)	0.143		
Tumor size	1.03 (0.99-1.07)	0.153		

### Predictive value of the RNETS for anti-PD-1 immunotherapy response

**Figure 7:**
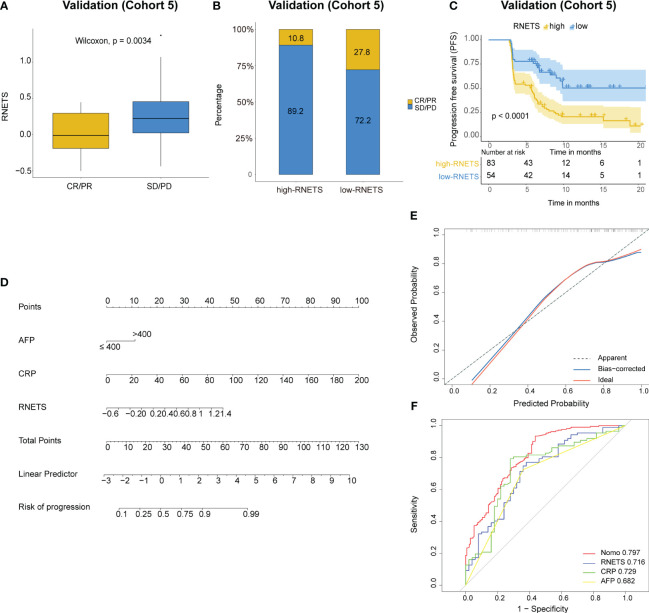
Predictive value of RNETS for anti-PD-1 immunotherapy response in cohort 5. **(A, B)** The treatment response to PD-1 inhibitors in high-RNETS and low-RNETS groups. **(C)** Kaplan-Meier curves of progression-free survival (PFS) for different RNETS groups. **(D)** Nomogram for predicting the risk of disease progression after PD-1 inhibitor therapy. **(E)** Calibration curves of the nomogram model. **(F)** Receiver operating characteristic (ROC) curves of progression status for the nomogram and three variables. CRP, C-reactive protein; AFP, a-fetoprotein; SD, stable disease; PD, progressive disease; PR, partial response; CR, complete response.

In cohort 5, the RNETS was significantly lower (mean: 0.0254) in the objective response group (CR/PR) than in the SD/PD group (0.2443) (**Figure 7A**). When dividing the patients into different RNETS groups with cut-off point of 0.0804, we found that the objective response rate (ORR) was significantly higher in the low-RNETS group than in the high-RNETS group (27.8% vs 10.8%) (**Figure 7B**). Moreover, 77.8% of patients in the low-RNETS group had disease control (PR, CR and SD). In the high-RNETS group, only 55.4% of patients had disease control (**Table 3**). Notably, Kaplan-Meier curve showed that the RNETS was significantly negatively associated with the PFS (*P*<0.001) (**Figure 7C**). These results indicated that the RNETS correlated with the clinical outcomes of anti-PD-1 immunotherapy. Multivariate Cox regression analyses revealed that RNETS, CRP levels, and AFP levels were independent prognostic factors for PFS (**Table 4**). To establish and validate a risk-scoring model for PFS, we created a nomogram integrating RNETS, AFP levels and CRP levels (**Figure 7D**). Calibration curve demonstrated the predicted value of disease progression was in accordance with the actual observed value (**Figure 7E**). Our results confirmed that the predictive value of the nomogram outperformed those of the individual RNETS, AFP levels and CRP levels for predicting PFS in patients receiving immunotherapy (**Figure 7F**). Subgroup analyses of treatment lines were performed to assess the performance of RNETS in evaluating the response to anti-PD-1 immunotherapy (**Figure S3**). Interestingly, patients with second-line therapy in low-RNETS group still had higher ORR (27.5% *vs* 10.3%, *P*=0.027) and longer PFS time (*P*=0.00016). Although there is currently not statistical significance, RNETS showed a gradually negative trend with immunotherapy benefit in first-line therapy subgroup (RNETS-L *vs* RNETS-H, ORR: 28.6% *vs* 12.0%, *P*=0.225; PFS analysis, *P*=0.077).

**Table 3:** The relationship between tumor response and RNETS groups in patients treated with PD-1 inhibitors.

	high-RNETS (n=83)	low-RNETS (n=54)	*P*
Treatment line (Immunotherapy)			0.595
First-Line	25 (30.1)	14 (25.9)	
Second-Line	58 (69.9)	40 (74.1)	
Treatment response			
**CR**	0 (0)	1 (1.9)	
**PD**	37 (44.6)	12 (22.2)	
**PR**	9 (10.8)	14 (25.9)	
**SD**	37 (44.6)	27 (50.0)	
**ORR(CR+PR)**	9 (10.8)	15 (27.8)	0.020
**DCR(CR+PR+SD)**	46 (55.4)	42 (77.8)	0.013

Variables are expressed as number of patients (%).

CR, complete response; DCR, disease control rate; ORR, objective response rate; PD, progressive disease; PR, partial response; SD, stable disease.

**Table 4:** Univariate and multivariate Cox regression analyses of risk factors for progression-free survival in Cohort 5.

	Univariate HR (95% CI)	*P* value	Multivariate HR (95% CI)	*P* value
AFP, ng/ml				
≤ 400	1.0			
> 400	2.71 (1.69-4.37)	<0.001	2.11 (1.29 - 3.48)	0.003
Age	0.98 (0.96-1)	0.040		
ALBI grade				
1	1.0			
2	0.96 (0.59-1.56)	0.868		
3	1.68 (0.84-3.39)	0.145		
Child-Pugh grade				
A	1.0			
B+C	1.09 (0.67-1.78)	0.728		
Cirrhosis				
Absent	1.0			
Present	1.56 (0.88-2.77)	0.128		
CRP	1.01 (1-1.01)	0.001	1.01 (1 - 1.01)	0.048
ECOG PS				
0	1.0			
1 + 2	0.94 (0.61-1.44)	0.766		
Embolus				
Absent	1.0			
Present	0.92 (0.6-1.42)	0.717		
Aetiology				
HBV	1.0			
HCV	1.33 (0.18-9.57)	0.779		
Others	1.11 (0.45-2.75)	0.817		
NLR	1.15 (1.06-1.25)	0.001		
RNETS	3.4 (1.86-6.2)	<0.001	2.49 (1.29 - 4.81)	0.007
Gender				
Female	1.0			
Male	0.84 (0.43-1.62)	0.599		
Tumor number				
≤ 3	1.0			
> 3	1.02 (0.65-1.59)	0.936		
Tumor size, cm	1.04 (0.99-1.09)	0.125		

## Discussion

As only a fraction of HCC patients have shown impressive efficacy to immune checkpoint blockade (30), there is an urgent need to discover robust predictive biomarkers for tracking patient’s response to immunotherapy in advanced HCC cases. Unfortunately, there are currently no reliable predictive biomarkers to support clinicians in predicting which patients would respond favorably or unfavorably to immunotherapy. Although PD-L1 and tumor mutation burden (TMB), are two of the most extensively studied predictive biomarkers for cancer immunotherapy (31–33), their predictive value in gauging patient responses to immunotherapy in HCC has been relatively limited (34, 35).

In cancers, the TIME acts as a major role in tumor metastasis, relapse, and resistance to treatment, due to which it has been under intensive investigation. To dissect TIME subtypes may help identify patients who would be likely to respond to immunotherapy. Since neutrophils are an integral part of the TIME, they could function as bridges between the tumor parenchyma and the immune microenvironment (36). Elevated neutrophil–lymphocyte ratio (NLR) in peripheral blood and high infiltration level of TANs in TIME are correlated with poor prognosis in HCC patients (37, 38). In addition, NETs, which are unique derivates of neutrophils, have been linked to progression and metastasis in certain solid tumors (10, 11, 39), and especially in HCC (12, 40). In this work, we have identified a constitutive NETs-related gene signature (NETS) to predict survival of HCC patients. We have noticed that these patients in high-NETS group were associated with poor prognosis.

Neutrophils are the most abundant immune cells in peripheral blood and play a fundamental role in inflammatory responses (41); however, their contribution to immune escape in malignancies is still controversial. Recent work has confirmed that TANs play an immunosuppressive role in primary liver cancer and that targeting TANs could be a potentially effective form of immunotherapy for treating liver cancer (42). In addition, the mechanism by which NETs promote immune escape is gradually becoming clearer; for example, it has been shown that NETs act as physical barriers that cover the cancer cells and shield them from immunotherapy (43). Studies have also shown that NETs can suppress T-cell responses to tumors by inducing metabolic and functional exhaustion in these immune cells (44). Our results also demonstrated that the NETS could be used to define TIME subtypes in HCC. Patients in the low-NETS group likely have an immunological ‘hot’ microenvironment, which may allow them to respond better to immunotherapy.

Using ELISA, IHC, and RNA-sequence data, it is possible to assess the abundance of NETs in peripheral blood and tumor tissues. However, these methods are expensive and complicated, which makes their large-scale application impractical. In China, CT examinations are widely used to diagnose liver cancer even in remote areas with underdeveloped medical resources. Therefore, using CT image-based radiological features for prognostics—especially if they reflect immune-related responses—will be of great clinical and economic value. In studies related to HCC, radiomics was usually used to predict clinical outcomes after surgery or responses to locoregional therapy (22, 23). However, most HCC patients already suffer from an advanced stage of cancer at the time of diagnosis and usually cannot opt for surgical treatment. For such HCC patients, immunotherapy can prolong their survival time and even help them reach a stage where translational surgery becomes a treatment option. In this study, we have developed a radiomics model-based score which we have named the RNETS, as a biomarker for clinicians to use for a quick prognostic indication of the immunological status of a tumor from CT images. In two internal HCC cohorts, the RNETS performed well enough to predict postoperative recurrence of the tumor and response to anti-PD-1 immunotherapy in HCC patients. The present study found that RNETS is associated closely with clinical benefit in patients received immunotherapy as second-line treatment. However, due to the limited number of first-line immunotherapy cohort, studies with a larger sample size should be implemented to further validate that RNETS is capable of predicting immunotherapy benefit for HCC patients, regardless of the therapy line.

There still remain some limitations in this study. First, the sample size in the training cohort from the TCIA database is rather small due to a scarcity of cases that have CT image information. Second, the number of NETs-related genes that have been identified is small and may be insufficient for a comprehensive assessment of NETs formation in TIME. Third, since serum NETs expression had been shown to be indirect means for inferring the NETs formation in tumor tissues, we believe that the results in this study will be more profound if we had integrated the information about NETs abundance in peripheral blood into overall analysis. Fourth, this study only covers NETs-related gene signature and tumor heterogeneity is enormous, future projects based on these results should be developed including various key pathway (for example, ferroptosis, hypoxia, epithelial-mesenchymal transition) related prognostic gene signatures and select the optimal radiomics model for implementation in clinical practice. Finally, this study was retrospective, and the results need to be validated using a prospective research framework.

In conclusion, we have developed a radiomics biomarker, the RNETS, which links NETs-related gene expression patterns in the TIME to radiomic features obtained from CT images. This scoring model can effectively and noninvasively predict the clinical outcomes and responses to immunotherapy in HCC patients.

## Data availability statement

The raw data supporting the conclusions of this article will be made available by the authors, without undue reservation.

## Ethics statement

The studies involving human participants were reviewed and approved by The Ethical Committee of Nanfang Hospital, Southern Medical University. Written informed consent for participation was not required for this study in accordance with the national legislation and the institutional requirements.

## Author contributions

HX and YPZ put forward the ideas of this article. HX, QL and YCZ were primarily responsible for conceptualization, methodology, and writing. JH, YS and MJL were responsible for data collection and data curation. JS, ML, MZ and WL were responsible for data revision. YB, YYZ and YPZ revised the manuscript. All authors contributed to the article and approved the submitted version.
