# Supplementary material for: Noninvasive evaluation of neutrophil extracellular traps signature predicts clinical outcomes and immunotherapy response in hepatocellular carcinoma
Source: Front Immunol. 2023 Jul 13;14:1134521. doi: 10.3389/fimmu.2023.1134521 (PMC10374215; doi:10.3389/fimmu.2023.1134521)
Supplement: Supplementary file 4 [file DataSheet_4.docx]

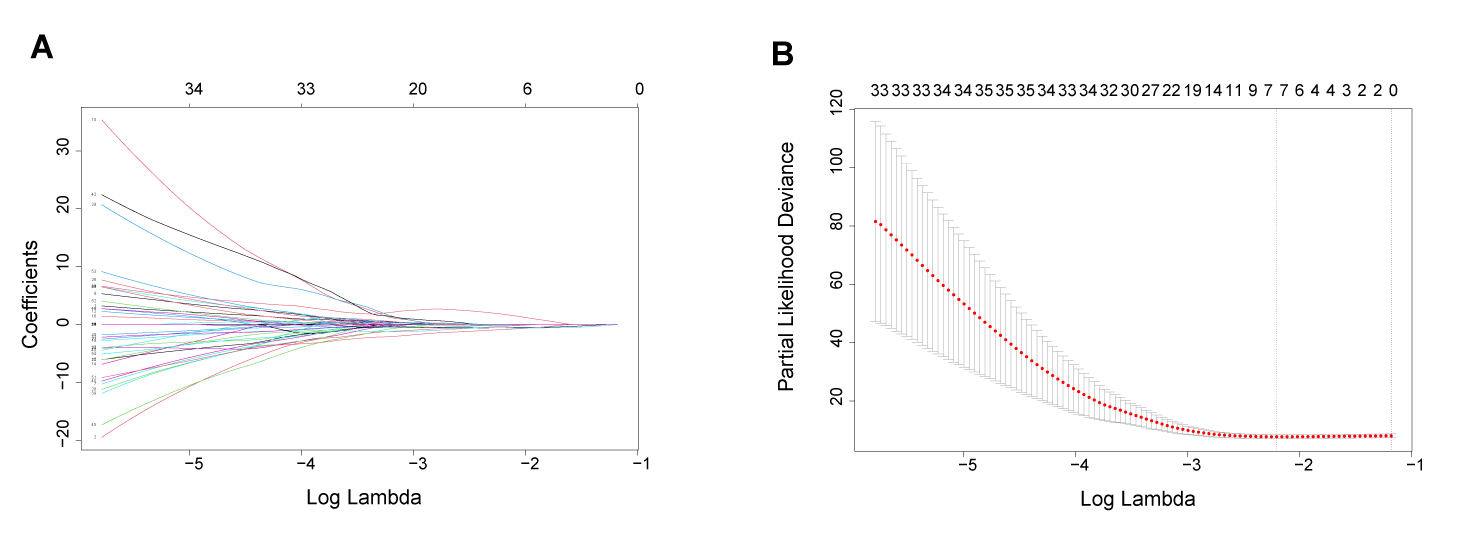


**Figure S1. (A)** LASSO coefficient profiles. **(B)** Partial likelihood deviance for the 6 NETs-related genes with 10-fold cross-validation.


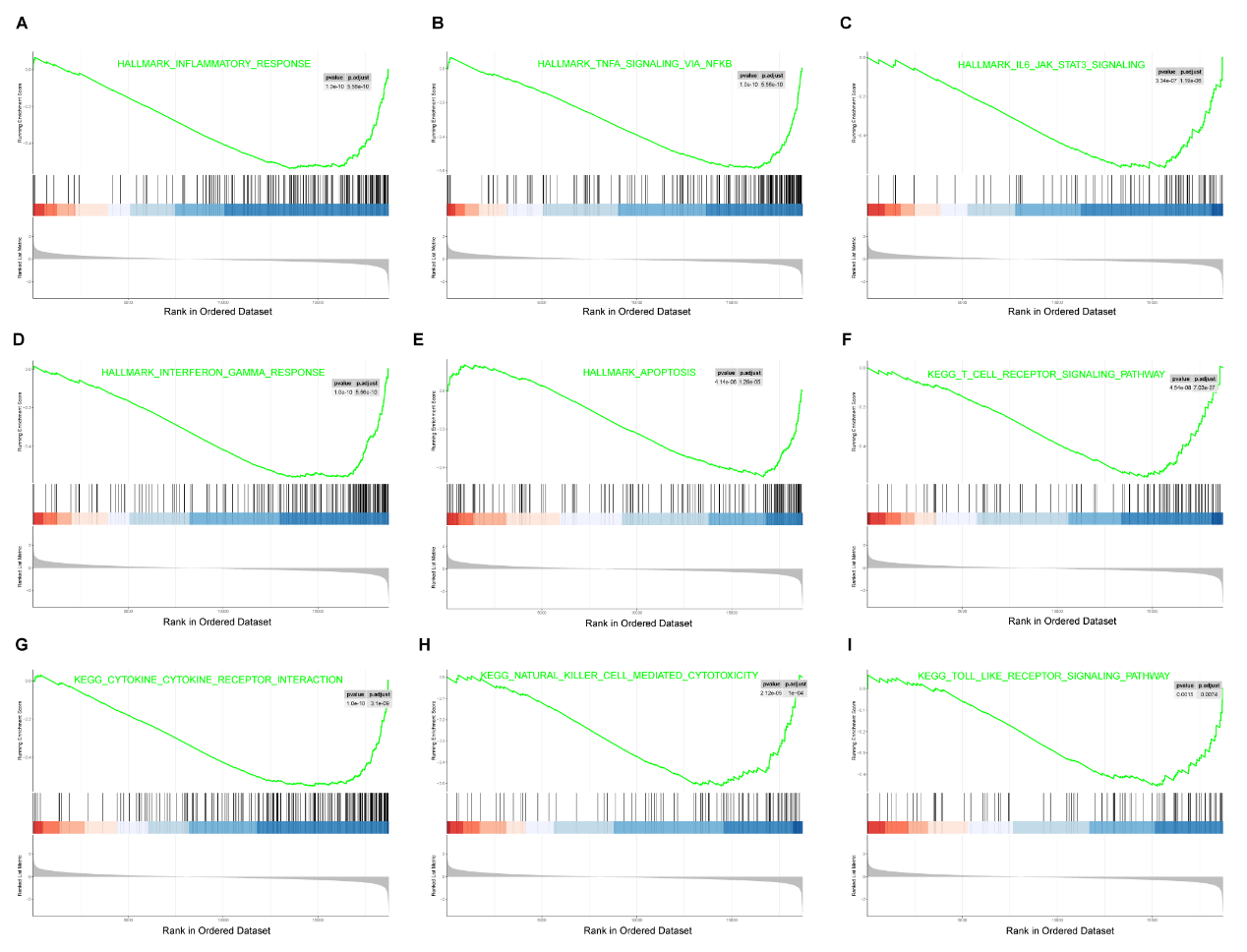


**Figure S2.** Potential molecular mechanism underlying the NETS. **(A-I)** Several immune response-related pathways identified in low-NETS group through gene set enrichment analysis (GSEA).


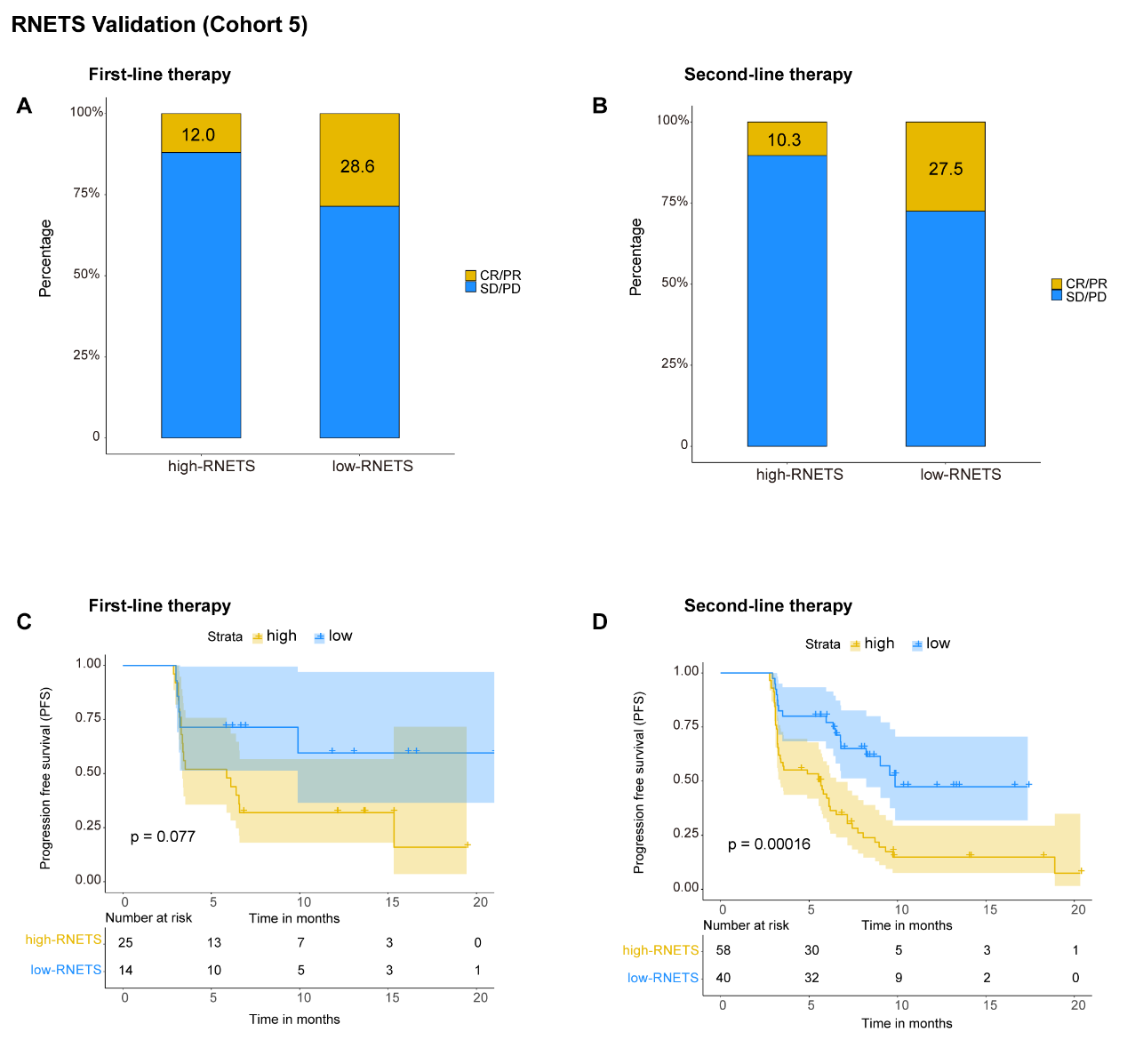


**Figure S3.** Evaluation of the response to anti-PD-1 immunotherapy in subgroup analysis. **(A)** The proportions of different responses to anti-PD-1 immunotherapy in patients who received immunotherapy as the first line treatment between RNETS-H and RNETS-L groups, **(B)** as the second line treatment. **(C)** Kaplan-Meier survival analysis of PFS for patients who received immunotherapy as the first line treatment, **(D)** as the second line treatment. PFS: progression free survival, RNETS-H: high-RNETS group, RNETS-L: low-RNETS group.
